# Utility of patient-derived xenografts to evaluate drug sensitivity and select optimal treatments for individual non-small-cell lung cancer patients

**DOI:** 10.1186/s10020-024-00934-4

**Published:** 2024-11-11

**Authors:** Xiaoqing Wang, Ju Zhu, Lingling Li, Qilin Zhao, Yutang Huang, Chunjie Wen, Dan Chen, Lanxiang Wu

**Affiliations:** 1 Pharmacogenetics and Pharmacogenomics Laboratory, School of Pharmacy, Chongqing Medical University, Chongqing, 400016 China; 2 Department of Cardiothoracic Surgery, The First Affiliated Hospital of Chongqing Medical University, Chongqing, 400016 China

**Keywords:** Non-small-cell lung cancer, Patient-derived xenografts, Drug sensitivity, Osimertinib resistance, Individualized chemotherapy regimen

## Abstract

**Background:**

Patient-derived xenograft (PDX) is currently considered a preferred preclinical model to evaluate drug sensitivity, explore drug resistance mechanisms, and select individualized treatment regimens.

**Methods:**

Histopathological examination, immunohistochemistry and whole-exome sequencing confirmed similarity between our PDX tumors and primary tumors in terms of morphology and genetic characteristics. The drug reactivity of the PDX tumor was validated in vivo. The mechanisms of acquired resistance to Osimertinib PDX tumors were investigated by WES and WB.

**Results:**

We successfully established 13 NSCLC-PDXs derived from 62 patients, including eight adenocarcinomas, four squamous-cell carcinoma, and one large-cell neuroendocrine carcinoma. Histological subtype and clinical stage were significant factors affecting the successful PDXs establishment. The treatment responses to conventional chemotherapy in PDXs were entirely consistent with that of their corresponding patients. According to the genetic status of tumors, more appropriate targeted agents were selected in PDXs for their corresponding patients as alternative treatment options. In addition, a PDX model with acquired resistance to osimertinib was induced, and the overactivation of RAS mitogen-activated protein kinase (MAPK)-extracellular signal-regulated kinase (ERK) signaling pathway caused by the dual-specificity phosphatase 6 (DUSP6) M62I mutation was found to play a key role in the development of osimertinib resistance. Trametinib, a specific inhibitor of the MAPK-ERK pathway significantly slowed down the tumor growth in osimertinib-resistant PDX models, providing an alternative treatment in patients after osimertinib failure.

**Supplementary Information:**

The online version contains supplementary material available at 10.1186/s10020-024-00934-4.

## Introduction

According to GLOBOCAN 2020, lung cancer is the second most diagnosed cancer and the leading cause of cancer deaths, accounting for approximately one in 10 diagnosed cancers (11.4%) and one in 5 deaths (18.0%) (Sung et al. 2021). Non-small-cell lung cancer (NSCLC) encompasses 80–85% of all lung cancer cases worldwide, and is further divided into three major subtypes—adenocarcinoma (ADC), squamous cell carcinoma (SCC), and large-cell carcinoma (LCC), with ADC being the most prevalent(Nicholson et al. 2022). The traditional treatments, including surgery, radiotherapy, and conventional chemotherapy, have shown limited efficacy in improving the outcomes of patients with NSCLC. Fortunately, identification of oncogenic driver mutations in ADC patients, as well as the rapid development of targeted therapeutic agents, such as epidermal growth factor receptor-tyrosine kinase inhibitors (EGFR-TKIs), have greatly enriched the arsenal against NSCLC and provided patients with better therapeutic outcomes and milder side effects (Wang et al. 2021; Liu et al. 2023). Nevertheless, only a minority of patients derive benefit from targeted therapy, primarily due to the relatively low frequency of known driver mutations in ADC patients. Additionally, there are currently no molecular-targeted agents proven effective against SCC and LCC (Boumahdi and Sauvage 2020; Kuribayashi et al. 2016). Even among responders, the drug resistance and cancer recurrence are great challenges in clinical treatment, and these patients need alternative treatment options, which hinge upon the emergence of new therapeutic targets (Meador and Hata 2020). During the past decade, tremendous efforts have been devoted to identifying new therapeutic targets and candidate treatment agents, but their effectiveness need to be accurately evaluated in pre-clinical models which can reflect the high diversity of histopathology and molecular genetics observed in NSCLC tissues.

In recent years, patient-derived xenograft (PDX) models have emerged as a powerful tool for the development of novel therapies for early, advanced, and drug-resistant tumors. These animal models are established by transplanting fresh tumor tissues resected from patients into immunodeficient mice. Therefore, PDX model accurately recapitulates the morphological, structural, and molecular characteristics of primary tumor, better reflecting the interaction from host microenvironment, gene expression patterns, and histological characteristics of the original patient when compared to standard cell line-derived xenograft (CDX) model(Zeng et al. 2023). To date, a series of PDXs have been developed and used as preclinical drug screening platforms, including for pancreatic cancer, colorectal cancer, lung cancer, esophageal cancer, and other cancers(Huang et al. 2020; Sorokin et al. 2022; Zou et al. 2018; Karkampouna et al. 2021; Sereti et al. 2018; Grunblatt et al. 2020). In addition to drug screening assays, PDXs can also be applied to study oncogenic signaling pathways, cancer progression and evolution, as well as the molecular mechanisms involved (Dobrolecki et al. 2016; Pardo-Sanchez et al. 2021). Therefore, it is a reliable strategy to provide guidance for optimizing personalized treatment in cancer and suggests new treatment opportunities for patients without other treatment options (Liu et al. 2023; Zeng et al. 2023).

Osimertinib is the first Food and Drug Administration (FDA)-approved third-generation EGFR-TKIs, and has been used as the first-line therapy for advanced EGFR-mutant NSCLC, regardless of T790M mutation status (Soria et al. 2018). Despite excellent clinical response, the majority of patients receiving osimertinib eventually develop acquired resistance. The elucidated molecular mechanisms of resistance are multiple and can be classified into EGFR-dependent and independent. However, in up to 55% of patients the underlying mechanisms remain unknown (Gomatou et al. 2023; Liao et al. 2023). Therefore, in-depth exploration of resistance mechanisms, development of alternative therapeutic options, as well as the appropriate preclinical tools to study the efficacy and toxicity of novel therapeutic strategies are urgently needed. PDX models are currently becoming a preferred research tool to optimize the development of anti-chemoresistance agents at multiple steps, including target exploration and validation, pharmacology, and translational studies (Schueler et al. 2019).

Here, we established a panel of NSCLC-PDXs by directly implanting tumor specimens collected from patients into immunodeficient mice. These PDX tumors preserved the histological and genetic characteristics of the primary tumors. Three representative patients and matched PDXs were selected to compare their responses to the same conventional chemotherapy regimens, and all PDXs closely recapitulated the clinical courses of their corresponding patients. According to the genetic status of tumors, we treated PDXs with new molecular-targeted agents, sotorasib and anlotinib, and found better efficacy and lower toxicity than that of the conventional chemotherapeutic drugs, indicating the more optimized treatments for their corresponding patients. Finally, we induced acquired resistance to osimertinib in PDXs derived from an ADC patient with EGFR L858R mutation, who was sensitive towards osimertinib treatment. Comparing the differences in gene status between osimertinib -resistant PDXs and sensitive PDXs, we found that the DUSP6 M62I mutation may cause overactivation of the RAS mitogen-activated protein kinase (MAPK)-extracellular signal-regulated kinase (ERK) pathway, leading to osimertinib resistance. Therefore, in cases where osimertinib is ineffective, alternative treatments such as trametinib, a specific inhibitor of this pathway, should be considered.

## Materials and methods

### Patients and tissue specimens

Sixty-two fresh tumor specimens were obtained from patients diagnosed with NSCLC before surgery in the Thoracic Surgery Department of the First Affiliated Hospital of Chongqing Medical University. Written informed consent was obtained from each patient. This study was approved by the ethics committee board of the First Affiliated Hospital of Chongqing Medical University (Approval number: 2022-K432), and was registered with the Chinese Clinical Trial Registry (No. ChiCTR2200066004). Clinical features of studied patients are summarized in Table S1. Immediately after surgery (within an average of 1 h after resection), tumor specimens were divided into 3 portions for implantation into immunodeficient mice, DNA/RNA extraction, and pathological assessment.

### PDX model establishment

All animal studies were carried out in accordance with the National Institutes of Health Guide for the Care and Use of Laboratory Animals, and approved by the Animal Ethics and Experimental Committee of the Chongqing Medical University. Nonobese diabetic/severe combined immune deficiency (NOD/SCID) female mice (6–8-week-old) were obtained from Changzhou Cavens Laboratory Animal Co., Ltd. (Changzhou, China). To establish the PDX model, five fresh tumor specimens (3–5 mm^3^ tumor specimens from different spatial regions of the primary tumor) were mixed and subcutaneously implanted into the flank of NOD/SCID mice under anesthesia, and collodion was applied around the skin incision for wound healing. For LCNEC tissue, 15 tumor specimens from different spatial regions of the primary tumor were implanted into three mice, with each mouse receiving five tumor specimens. The tumor size was measured with calipers once a week and tumor volume were calculated by using the formula: Volume (mm^3^) = (length × width^2^)/2. When the tumor size was > 1 cm^3^, the PDX mice were anesthetized with 3% pelltobarbitalum natricum and the tumors were further implanted into another cohort of mice, frozen for molecular and histological analysis. Engraftment success was defined as completion of the transfer to the third generation.

### Histological staining

Surgically resected tumors and PDX tissues were formalin fixed and embedded in paraffin, cut into 4 μm thick sections. H&E staining was used for assessment of pathology. For IHC, sections were treated with primary antibodies against human NapsinA (1:100, ZSGB-Bio), human TTF1 (1:100, ZSGB-Bio), human p63 (1:100, ZSGB-Bio), human CD56 (1:400, CST), human chromogranin (1;300, HuaBio), and human synaptophysin (1:400, Abcam), human ERK(1:100, ZEN-BIOSCIENCE), human p-ERK(1:100, ZEN-BIOSCIENCE) at a temperature of 4 °C overnight. After washing three times with PBS, sections were incubated at room temperature with biotinylated secondary antibody (Vectastain ABC Kit, Vector Laboratories, CA) for 30 min. Pathological examinations were performed under light microscopy by two pathologists blinded to the clinical information of patients. For immunohistochemistry analysis, light levels were adjusted to preset values before image acquisition to ensure data fidelity. Image J software was utilized to analyze area and Integrated Optical Density of all detectable positively labeled cells and calculate the Average Optical Density (AOD) for quantitative analysis according to the formula: AOD = Integrated Optical Density / Area. Each group contains three tumors. From each tumor, five slices are selected from different parts, and three representative fields of view are taken from each slice.

### WES analysis

Genomic DNA from NSCLC primary tumors and PDX tumors was extracted using Gen Elute MammalianGenomic DNA Miniprep kits (Sigma-Aldrich, USA), and was used for WES library construction. Briefly, approximately 3 μg genomic DNA was sheared to 150–220 bp fragments using sonicator (Covaris, MA). The sheared deoxyribonucleic acid (DNA) was purified using the Agencourt AMPure XP kit (Beckman Coulter, USA). Adapters from Agilent were ligated onto the polished ends and the libraries were amplified by polymerase chain reaction (PCR). The exome capture was performed using the Agilent SureSelect Human All Exon V6 (Agilent Technologies, Santa Clara, CA) according to the manufacturer’s instruction. The DNA fragments bound with the probes were washed and eluted with the buffer provided in the kit. Then these libraries were sequenced on the Illumina sequencing platform (HiSeq X-10, Illumina, Inc., CA) and 150 bp paired-end reads were generated. The target coverage of the captured region was 100x. The initial raw data were in fastq format and underwent preprocessing using fastp (Version: 0.19.5), which included adapter trimming and quality control. For WES data from PDX tumors, we applied an additional step to filter out mouse-originated reads using DeconSeq (version. 0.4.3) with the reference genomes of human (GRCh37) and mouse (GRCm38). We kept the human-specific reads only for subsequent analyses. The resulting clean reads were then aligned to the reference human genome (GRCh37) using BWA (version 0.7.12). The mapped reads were sorted and indexed by using SAMtools (Version 1.4). GATK (Version 4.1.0.0) was utilized for recalibrating the base quality score and for realigning single nucleotide polymorphisms (SNPs) and insertion/deletion (INDELs), while Picard (Version 4.1.0.0) was employed to mark duplicate reads, resulting in the generation of analysis-ready BAM files. These final BAM files served as input for variant calling. During SNP and INDEL calling, numerous annotation databases, including RefSeq, 1000 Genomes, the Catalogue of Somatic Mutations in Cancer (COSMIC), and OMIM, were consulted and annotated using ANNOVAR.

### Epstein-Barr virus (EBV) detection assay

The presence of EBV specific RNA transcripts was determined by in situ-hybridization (ISH). EBER oligonucleotides were added to formalin-fixed paraffin-embedded tissue sections and detected using Epstein-Barr Virus (EBER) PNA Probe/Fluorescein and PNA-ISH Detection Kit (Dako, Denmark) according to the manufacturer's instructions.

### Preclinical efficacy of chemo- and targeted therapies in PDXs

The PDXs derived from Patient #18, #21, and #19 were used to test the response to the conventional and targeted chemotherapeutic drugs. In brief, the conventional chemotherapeutics paclitaxel (7.5 mg/kg), carboplatin (25 mg/kg), etoposide (30 mg/kg), and nedaplatin (10 mg/kg) were administered intravenously into PDX mice every 5 days with a total of 4 dose, respectively. The molecularly targeted agents sotorasib (10 mg/kg), anlotinib (3 mg/kg) and trametinib (0.3 mg/kg) were administered to PDX mice via oral gavage every 2 days with a total of 10 dose, respectively. Each treatment arm contained a minimum of three PDX replicates. Mice in the control group received an equivalent volume of phosphate buffered saline (PBS). The treatment response was evaluated by measuring the tumor volume of mice. As previous reported, we defined PDX response as > 20% tumor shrinkage after treatment, no response as > 20% growth after treatment, and stable disease otherwise (Stewart et al. 2015). Response in patients was evaluated based on RECIST version 1.1 (Eisenhauer et al. 2009).

### Construction of PDX model with acquired resistance to osimertinib

The PDXs derived from Patient#42 were used to establish osimertinib-resistant model. Briefly, the P3 generation of PDXs were passaged into six NOD/SCID mice at the age of 6–8 weeks. When the tumor volume reached approximately 100 mm^3^, PDX mice were randomly divided into osimertinib group and control group, with three mice in each group. Mice in the osimertinib group received 10 mg/kg per day orally by gavage, while control mice received an equivalent amount of PBS. Tumors were measured twice a week, until the volume of three consecutive measurements increased by 10% compared to the volume of the previous measurement, at which point drug resistance induction was considered successful.

### Establishment of patient-derived cells

Fresh PDX tumors were collected and subsequently conditioned in ice-cold PBS with 10 mM HEPES and 100 U/mL penicillin–streptomycin (Thermo Fisher Scientific). Necrotic regions and adipose tissue were excised wherever feasible. The tissues were then minced into small pieces and digested in 5 ml of PBS/EDTA (1 mM) containing collagenase I (Thermo Fisher Scientific) at a concentration of 200 U/mL of the enzyme for a period of one hour. The dissociated cells were collected using 40 mm filters. Following a 10-min centrifugation at 300 g and 4 °C, the cell pellets were re-suspended in medium and seeded at a concentration of 10^5^ cells/cm^2^. The cultures were maintained in an incubator at 37 °C with 5% CO_2_ and the cell growth medium was refreshed every 2–3 days. Once the cells were confluent, they were digested with 0.05% EDTA-trypsin for passage. Cells were stained with a human EpCAM antibody (Miltenyi Biotec, Bergisch Gladbach, Germany) according to the manufacturer’s instructions. Flow cytometry was performed using FACSVerse (BD Biosciences, California, USA) and analyzed with FlowJo software. The isolation of primary tumour cells is deemed successful when the number of EpCAM-positive cells exceeds 80% (Figure S1).

### Cell culture and transfection

The PC9 and H1975 NSCLC cell lines were acquired from the American Type Tissue Collection (Manassas, VA). They were cultured in RPMI 1640 medium supplemented with 10% FBS and 1 × Antibiotic/Antimycotic. All experiments were initiated during the logarithmic growth phase. For transfection of cell plasmids, either wild-type DUSP6 plasmids or mutated plasmids (Youbio Biotechnology) were transiently transfected into PC9 or H1975 cells using Lipofectamine 2000 reagent (Beyotime Biotechnology) following the manufacturer’s protocol. The protein expression level was determined by immunoblotting assay after culturing for 48 h.

### Cell viability assay

According to the manufacturer’s instruction, cell viability was determined using the Cell Counting Assay Kit-8 (CCK-8) (Beyotime Biotechnology). Briefly, differently treated cells were seeded into 96-well plates at a density of 5000 cells/well. CCK-8 was added to the culture medium at the indicated time points, and then the cells were incubated for another 2 h. The optical densities at 450 nm were detected, and the absorbance was used to indicate cell survival. For the cytotoxicity assay, the cells were pretreated with serial doses of osimertinib, and the inhibition concentration (IC50) of this chemotherapeutics was determined using probit analysis.

### Western blot analysis

We extracted the total protein of the cells using RIPA lysis buffer (Beyotime, China). Briefly, a total of 20 µg of protein per sample was separated by 10% SDS/PAGE and transferred to PVDF membranes. The membranes were first incubated for 2 h at room temperature in 5% BSA and then overnight at 4 °C with primary antibodies against ERK1/2 (1:1000; ZEN-BIOSCIENCE), p-ERK1/2(1:1000; ZEN-BIOSCIENCE), DUSP6(1:1000; Huabio), GAPDH(1:10000; ZEN-BIOSCIENCE), followed by incubation with secondary antibodies (1:1000; Beyotime) conjugated with horseradish peroxidase at room temperature for 1 h. The protein bands were detected by using an enhanced chemiluminescence plus kit (Millipore, USA) as recommended by the manufacturer. The Western blot signals were quantitated by densitometric analysis using ImageJ.

### Statistical analysis

All the graph, calculation, and statistical analyses were generated using GraphPad Prism software version 8.0 for Windows (GraphPad Software, USA). To determine clinical parameters that contributed to the establishment of PDXs, the Fisher's exact test was conducted to evaluate the correlation between success rates and clinical pathological parameters. For the therapeutics study, data were analyzed by two-way ANOVA analysis of variance for multiple comparisons, followed by Dunnett’s test for comparisons between two groups. These data represent at least three independent experiments and are expressed as the means ± SD. A value of *P* < 0.05 was considered statistically significant.

## Results

### Histological subtype and clinical stage were affecting the PDX establishment

**Fig. 1:**
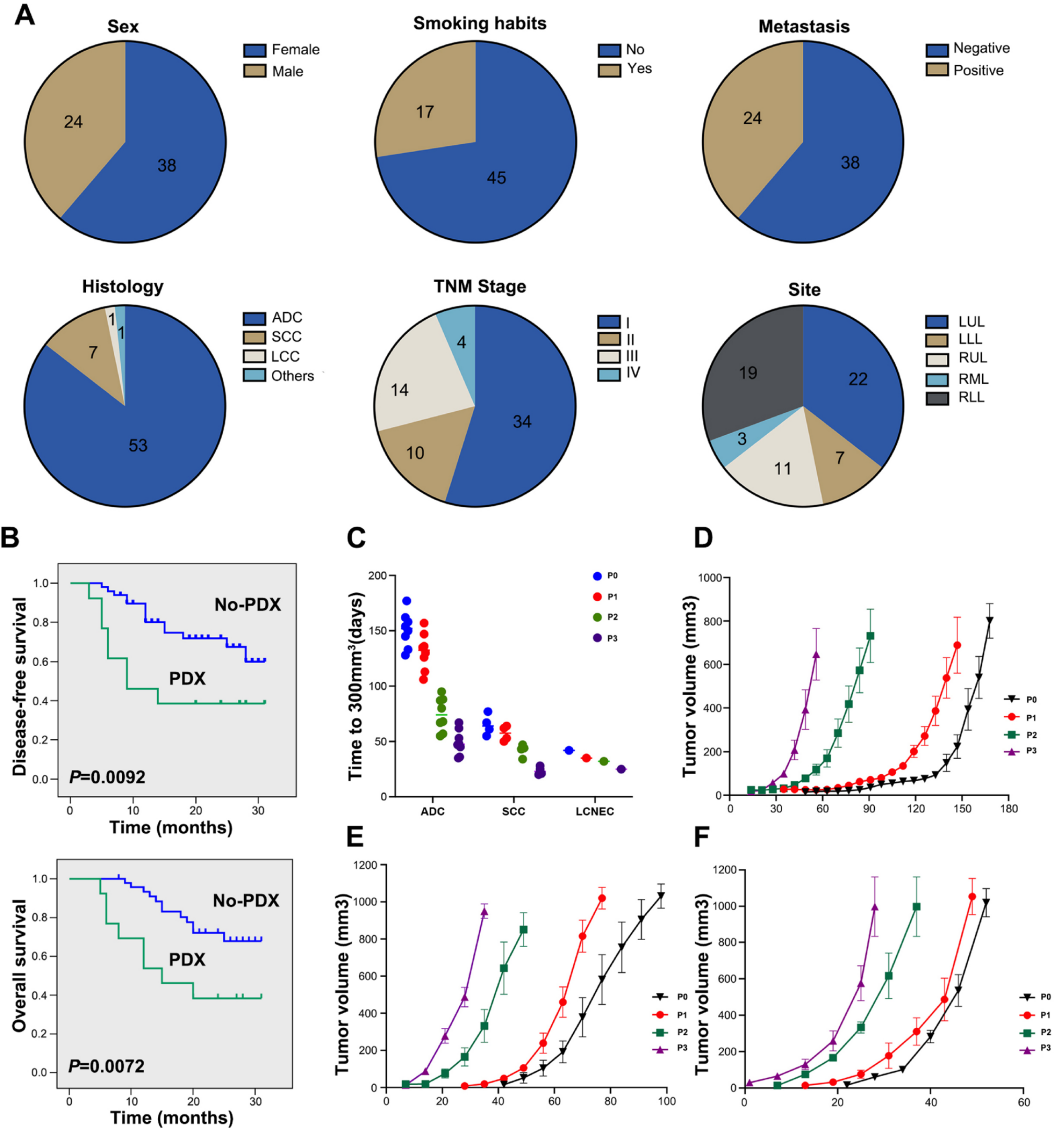
Clinicopathological characteristics of NSCLC patients and PDX tumor growth characteristics. **A** Clinicopathological characteristics of NSCLC patients. ADC, adenocarcinoma; SCC, squamous cell carcinoma; LCNEC, Large cell neuroendocrine carcinoma; LUL, left upper lobe; RLL, right lower lobe; RML, right middle lobe; LLL, left lower lobe; RUL, right upper lobe. **B** Kaplan–Meier curves for DFS and OS of NSCLC patients according to the engraftment status of their corresponding PDXs. Kaplan–Meier survival analysis and log-rank test were used to perform the survival analysis (No-PDX: *n* = 49; PDX: *n* = 13). **C** The scatter plot represents the time to reach 300 mm^3^ tumor volume in mice; each point represents the time taken for a tumor to reach 300mm^3^. **D** Growth curves of PDX engrafted tumors from 9 ADC patients. **E** Growth curves of PDX engrafted tumors from 4 SCC patients. **F** Growth curves of 3 PDX engrafted tumors from one LCNEC patient

In this study, we collected 62 tumor samples from 62 different patients with newly diagnosed NSCLC, who had not been previously treated. The average age of patients was 63.7 years, ranging from 44–79, 61% were females (38 out of 62 cases), and 27% were smokers (17 out of 62 cases). Most of the patients (71%, 44 out of 62 cases) were diagnosed in early stages of the disease (34 cases in stage I, 10 cases in stage II); and 85% had ADC (53 out of 62 cases), with acinar, mucinous, and solid ADC being the most prevalent subtypes. The primary tumors of these patients originated from various lobes of the lung, among which the left upper lobe (LUL) was the most involved, while the right middle lobe (RML) was the least (Fig. 1A).

The generation harboring the patient-derived tumor tissue was termed P0, with subsequent generations numbered consecutively (P1, P2, P3 and so on). Engraftment success was de-fined as completion of the transfer to the third generation. Of the 62 engraftments, 13 led to the successful establishment of PDXs, which included eight ADCs, four SCCs, and one large-cell neuroendocrine carcinoma (LCNEC), representing a tumor take rate of 21% (Table 1). Additionally, lymphoproliferations occurred in 3 (4.8%) xenografts, despite none of these patients having a prior or subsequent clinical history of lymphoproliferative disease. As EBV-associated lymphoma occurs in immunocompromised patients and transplant recipients, we performed in situ hybridization for EBV-encoded small RNAs (EBER) to assess the EBV status of these lymphoproliferations (Shannon-Lowe et al. 2017). All lymphoproliferations were positive for EBER and human CD20 antigen, indicating these tumors were EBV-associated human diffuse large B cell lymphoma which was formed by clonal proliferation of human B-cell lymphocytes. In addition, the liver and spleen of these mice were significantly larger than normal mice (Figure S2). Previous reports have shown that a small portion of PDXs may undergo a transformation during passage processes, resulting in the engraftments are of lymphocytic, rather than tumor origin (Pearce et al. 2023; Williams et al. 2019). Therefore, they were excluded from subsequent analysis. Next, we analyzed the relationship between engraftment rate of specimens and clinicopathological parameters in NSCLC patients, and found that histological subtype and clinical stage were significant factors affecting the PDXs engraftment. The success rate of ADC (8/53; 15%) was remarkably lower in comparison with the other subtypes (SCC, 4/7, 57%; LCNEC, 1/1, 100%), and the advanced stage (III/IV; 7/18, 39%) was linked to higher chance of a successful engraftment compared with the early stage (I/II; 6/44, 13.6%). However, other factors, including age, sex, smoking status, primary tumor size, and lymphatic metastasis, did not correlate with the engraftment rate (Table 2). Moreover, patients with successful tumor engraftment had a significantly shorter DFS and OS than those without establishment of PDX (Fig. 1B). Additionally, we observed the growth characteristics of the successfully implanted tumors, PDX tumors were passaged three generations in mice (P1-3), in addition to the original xenograft (P0). Our results showed that the time required for grafts from different patients to grow to 300 mm^3^ fluctuated between 42 and 177 days in P0 generation, with the average growth time of 116 days. However, the latency time of the subsequent passages became shorter, and the average growth time decreased to 101, 61, and 39 days in P1, P2, and P3 generation, respectively (Fig. 1C). In addition, the growth curves of xenografts from the same tumor tissue in specific passages were similar, but not entirely consistent, which might be due to the heterogeneity of tumor cells from a single tumor tissue and the interindividual difference in the immune reactivity of mice. However, the growth curves of xenografts from different pathological tissues in specific passages were significantly different. Among them, LCNEC showed the fastest growth, followed by SCC and ADC (Fig. 1D-F).

**Table 1:** Clinical and pathological characteristics of 13 NSCLC patients

Patient ID	Sex	Age (years)	Smoking habits	Histology	TNM	Stage	Site	Tumor size (cm3)	Metastasis	Driver oncogene status
Patient#1	Female	50	No	ADC	T2N3M1	IVA	LLL	13.50	Yes	ALK^+^
Patient#3	Female	64	No	SCC	T1bN0M0	IA2	RLL	1.52	No	WT
Patient#9	Male	68	Yes	ADC	T1bN2M0	IIIA	RUL	5.78	Yes	WT
Patient#18	Female	69	No	ADC	T2aN2M0	IIIA	LUL	12.80	Yes	KRAS G12C
Patient#19	Male	66	Yes	LCNEC	T2aN0M0	IB	LLL	13.50	No	WT
Patient#21	Male	58	Yes	SCC	T2aN0M0	IB	RLL	14.60	No	WT
Patient#30	Female	56	No	ADC	T1cN0M0	IA3	LLL	19.60	No	EGFR exon 21 L858R
Patient#42	Female	78	No	ADC	T1cN2M0	IIIA	RUL	27.20	Yes	EGFR exon 21 L858R
Patient#43	Female	56	No	ADC	T1cN0M0	IA3	LUL	27.40	No	WT
Patient#44	Female	44	No	ADC	T4NXM1a	IVA	LUL	27.80	Yes	EGFR exon 19 del
Patient#46	Male	63	Yes	SCC	T2aN1M0	IIB	LLL	29.30	Yes	KRAS G13C
Patient#49	Male	63	Yes	SCC	T4N0M0	IIIA	LUL	30.34	No	WT
Patient#50	Female	59	No	ADC	T2N2M0	IIIA	LUL	31.97	Yes	EGFR exon 21 L858R

Driver oncogene: The American Society of Clinical Oncology (ASCO) advocates for routine mutation testing of driver genes, including EGFR, ALK, ROS1, and BRAF, in patients with non-small cell lung cancer (NSCLC)

*TNM* tumor node metastasis, *ADC* adenocarcinoma, *SCC* squamous cell carcinoma, *LCNEC* Large cell neuroendocrine carcinoma, *LUL* left upper lobe, *RLL* right lower lobe, *RML* right middle lobe, *LLL* left lower lobe, *RUL* right upper lobe

**Table 2:** Correlation between clinical characteristics of NSCLC patients and establishment of PDXs

Variables	Engrafting	Non-engrafting	Total	Established rate (%)	*P* value
Engraftment	13	49	62	21.0%	
Age					
≤ 60	6	18	24	25%	0.541
> 60	7	31	38	18.4%	
Sex					
Female	8	30	38	21.1%	> 0.999
Male	5	19	24	20.8%	
Smoking status					
Never	8	37	45	17.8%	0.319
Former/ current	5	12	17	29.4%	
Histology					
ADC	8	45	53	15.1%	0.042*
SCC	4	3	7	57.1%	
LCNEC	1	0	1	100%	
Others	0	1	1	0	
Stage					
I/II	6	38	44	13.6%	0.040*
III/IV	7	11	18	38.9%	
Tumor size (cm^3^)					
< 11 cm^3^	2	14	16	12.50%	0.484
≥ 11 cm^3^	11	35	46	23.91%	
Metastasis					
Negative	6	32	38	18.8%	0.222
Positive	7	17	24	29.2%	
Site					
left lung	9	20	29	31.03%	0.116
Right lung	4	29	33	12.12%	

^*^Fisher’s exact test

### PDX tumors preserve morphologic and genetic features of the primary tumors

**Fig. 2:**
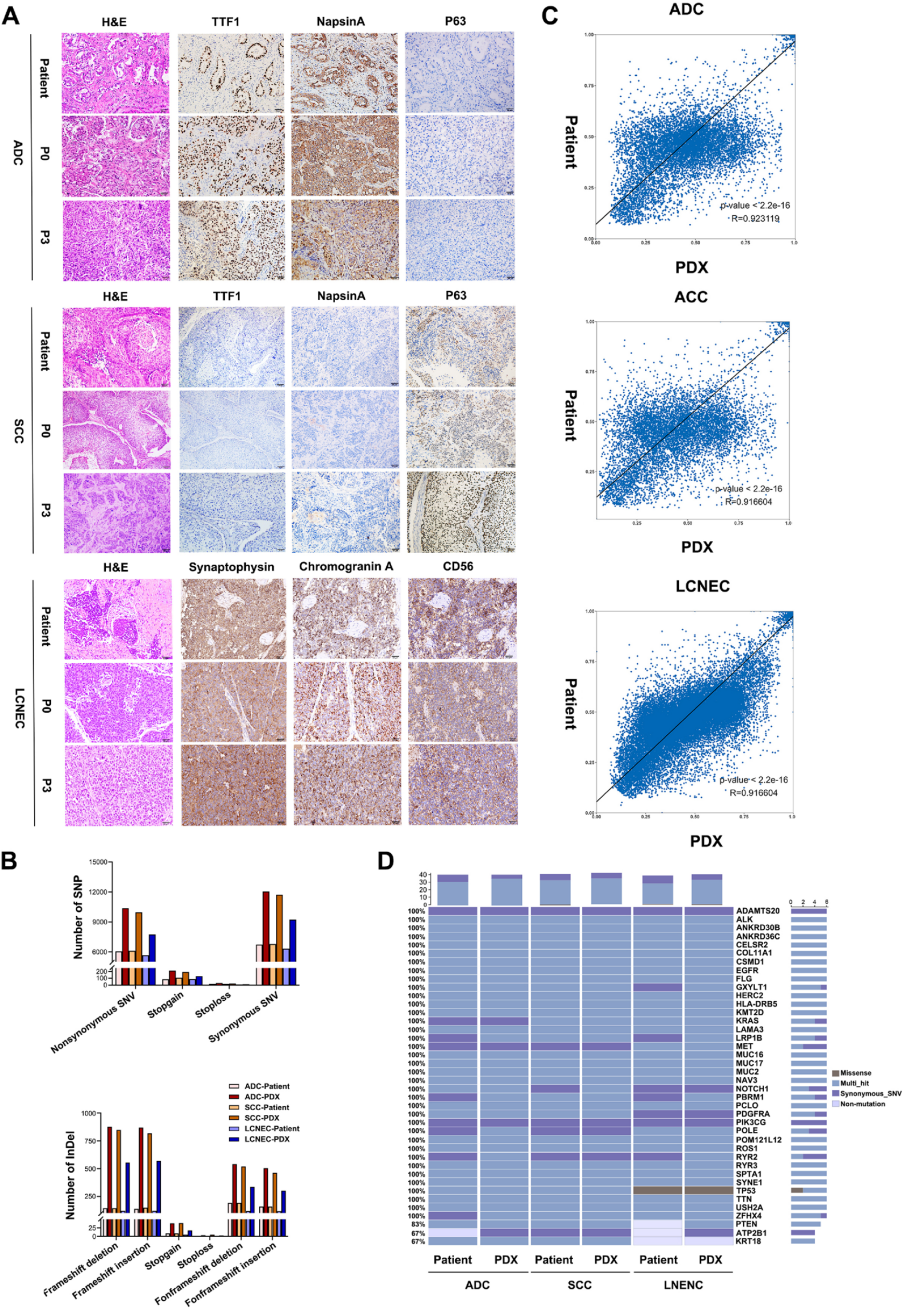
Morphologic and Genetic characteristics of the primary tumors and corresponding PDX tumors. **A** Representative images of the primary tumors and corresponding PDX tumors. Scale bar, 50 μm. **B** The total number of mutated genes detected in three pairs of tumors, the left graph illustrates the count of SNPs, while the right graph illustrates the count of InDels, all mutations on a single gene are counted only once. **C** Comparison of gene mutation similarity between the primary tumors and paired P3 generation PDX tumors, variant allele frequencies (VAF) of major mutations identified in both patient and PDX tumors were analyzed. Sites exhibiting no mutation or multiple mutated bases in both samples were excluded from further analysis. Additionally, sites with a Total Depth (DP) below 30 in either sample were also excluded. **D** Mutation status of 40 cancer-associated genes were compared between the primary tumors and corresponding PDXs

To evaluate whether the established PDXs could retain histological characteristics with the primary tumors from patients, we performed histopathological and immunohistochemical (IHC) examinations using all successfully grafted PDX tumors and their corresponding patient tissues. Tumor sections were stained with hematoxylin and eosin (H&E), and immune-stained for clinically relevant biomarkers, including the primary markers for ADC (TTF1 and Napsina), SCC (P63), as well as LCNEC (CD56, Synaptophysin, Chromogranin). H&E staining revealed that the PDX tumors were typically poorly differentiated and lacked certain structural features observed in the primary tumors, such as the acinus and nipple formations seen in ADC, as well as the keratinized structure present in SCC. This indicates that the poorly differentiated components are more prone to tumor formation in the PDX model. Nevertheless, tumor cell morphology such as irregular cellular patterns and heterogeneous nuclei remained similar to the primary tumor, and the adenocarcinoma cytoplasm was similarly heterogeneously stained. Additionally, the expression of biomarkers was positive and coincident in patient and mice tissues, which maintained over multiple passages (Fig. 2A). The results showed that the morphology and immunophenotype of PDX tumors were similar to primary tumors.

To further investigate whether the PDXs preserved the genetic profiles of the primary tumors, we performed whole exome sequencing (WES) analysis on tumors from three representative patients (one ADC (Patient#18), one SCC (Patient#21), and one LNENC (Patient#19)) and their corresponding PDX tumors (P3 generation). The number of different types of SNPs and InDels mutations detected in primary tumors and PDX tumors is shown in Fig. 2B. Compared to patient tumors, the detected number of single nucleotide polymorphisms (SNPs), insertions, and deletions (INDELs) in the matched PDX tumors were dramatically increased. A total of 18,335, 18,449, and 24,731 SNPs were detected in the tumors of Patient#18, Patient#21, and Patient#19, respectively. However, 31,175, 30,122, and 30,739 SNPs were detected in their matched PDX tumors. Similarly, 1,906, 1,945, and 4,285 INDELs were detected in the tumors of Patient#18, Patient#21, and Patient#19, respectively, while 6,015, 5,662, and 6,689 INDELs were detected in their matched PDX tumors. Approximately 88–99% of SNPs found in the patient tumors were retained in their matched PDXs. Correlation analysis indicated the similarity of SNV mutation in each pair of PDX and their corresponding primary tumor, with R-values all greater than 0.91(Fig. 2C). Mutations in 40 cancer-associated genes were generally preserved between PDX and patient tumors (Chakravarty et al. 2017; Rhodes et al. 2007; Ananda et al. 2015)(Fig. 2D). Together, these results show that PDXs retain the genetic profiles of their primary tumors.

### PDX model guided the selection of potentially effective therapy in NSCLC

We have shown the histological and genetic consistence between PDX model and patient.

To further assess the value of our PDXs in clinical individualized treatment for NSCLC patients, three typical NSCLC patients (Patient#18, Patient#21, and Patient#19) with established corresponding PDXs (P3 generation) were evaluated. Patient#18 was a 69-year-old non-smoking female, computed tomography (CT) revealed a 3.4*1.8 cm mass in the left upper lobe, which was pathologically diagnosed as low-differentiated ADC (T2aN2M0, stage IIIA), accompanied by Kirsten RAS (KRAS) G12C mutation. Patient#21 was a 58-year-old smoking male, CT results showed a 3.1*2.6 cm mass in the right lower lobe. Pathological examination showed a moderately differentiated SCC (T2aN0M0, stage IB). After surgery, both patients received 4 cycles of paclitaxel plus carboplatin treatment, and achieved clinical complete response. Patient#19 was a 66-year-old smoking male, CT scan demonstrated a mass of 2.6*2.2 cm in the left lower lobe, and the pathological diagnosis was LCNEC (T2aN0M0,IB stage). After surgery, this patient received 6 cycles of etoposide plus nedaplatin treatment, and achieved clinical complete response (Figure S3).

**Fig. 3:**
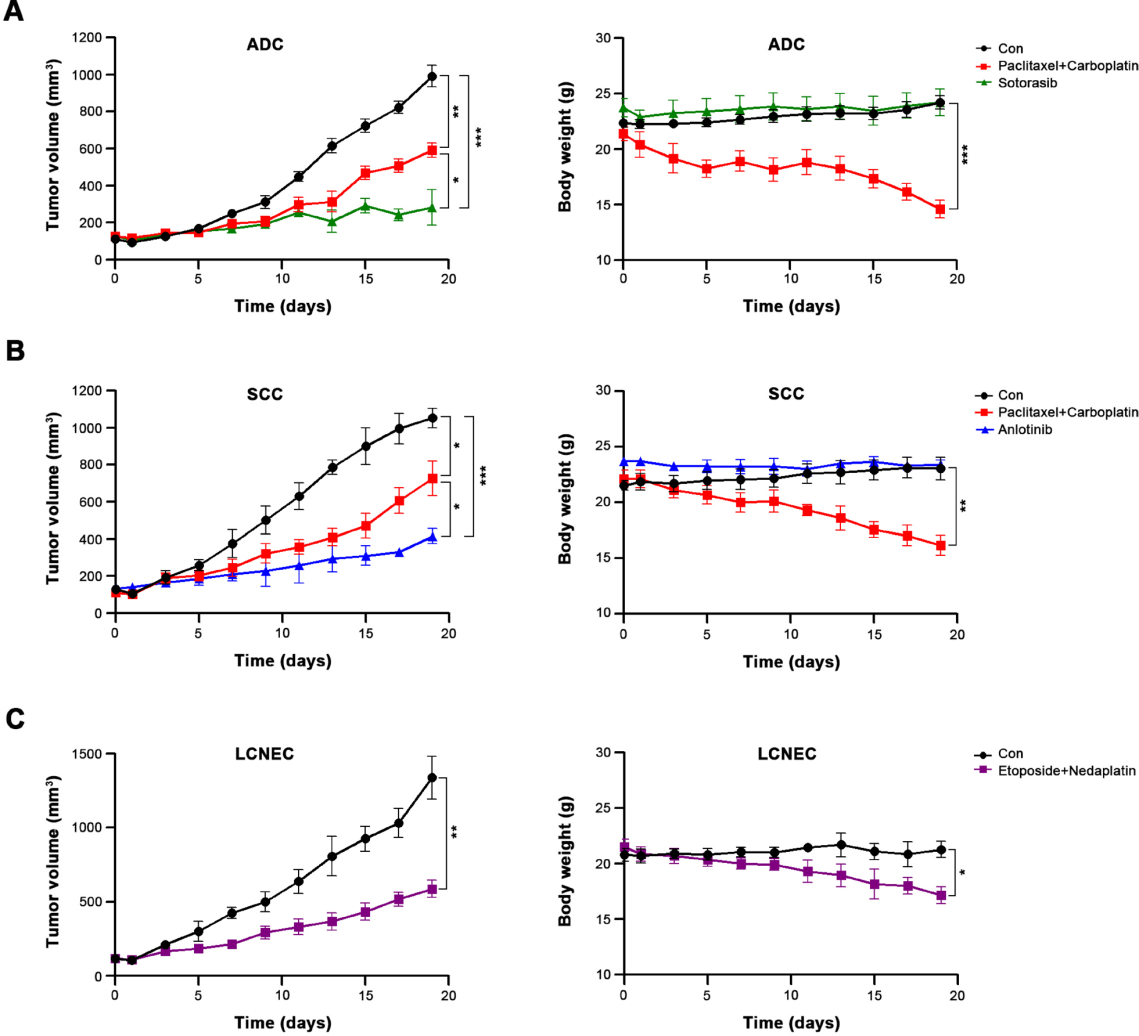
Efficacy validation of conventional and targeted chemotherapeutic agents in PDXs. **A** The curves for tumor volume and mice body weight of PDXs derived from Patient#18 after treatment with paclitaxel + carboplatin or sotorasib. **B** The curves for tumor volume and mice body weight of PDXs derived from Patient#21 after treatment with paclitaxel + carboplatin or anlotinib. **C**The curves for tumor volume and mice body weight of PDXs derived from Patient#19 after treatment with etoposide + nedaplatin. Differences in tumor growth between treatment groups were evaluated using two-way ANOVA, followed by Dunnett’s test for comparisons between two groups. Each group contains three mice, *P < 0.05, **P < 0.01, ***P < 0.001

In PDXs, we first validated the therapeutic efficacies of abovementioned conventional chemotherapy regimens, and found that both treatments significantly inhibited the growth of PDX tumors, with the responses were entirely consistent with those of their corresponding patients. However, the mice experienced significant weight loss, especially in ADC and SCC groups, which indicated a high toxicity of these conventional chemotherapeutic drugs. Therefore, we simultaneously investigated the efficacy and toxicity of sotorasib and anlotinib in the ADC and SCC PDX, respectively, based on the genetic status of tumors. Sotorasib is a novel KRAS-G12C inhibitor, and approved for the treatment of adult patients with KRAS G12C-mutated locally advanced or metastatic NSCLC who have received at least one prior systemic therapy (Blair 2021). Anlotinib is a multiple TKI and approved for the treatment of patients with locally advanced or metastatic NSCLC who have undergone progression or recurrence after ≥ 2 lines of systemic chemotherapy (Syed 2018). As expected, these two targeted drugs showed dramatically better efficacy and lower toxicity compared with the conventional chemotherapeutic agents, and were the optimized treatments for these two patients (Fig. 3). These results indicate that the treatment response of PDXs is similar to the clinical results. What’s more, the application of sotorasib and anlotinib in PDX model provided newly insight in the preclinical evidence for NSCLC treatmen.

### The involvement of MAPK-ERK signaling pathway in acquired resistance to osimertinib in NSCLC

**Fig. 4:**
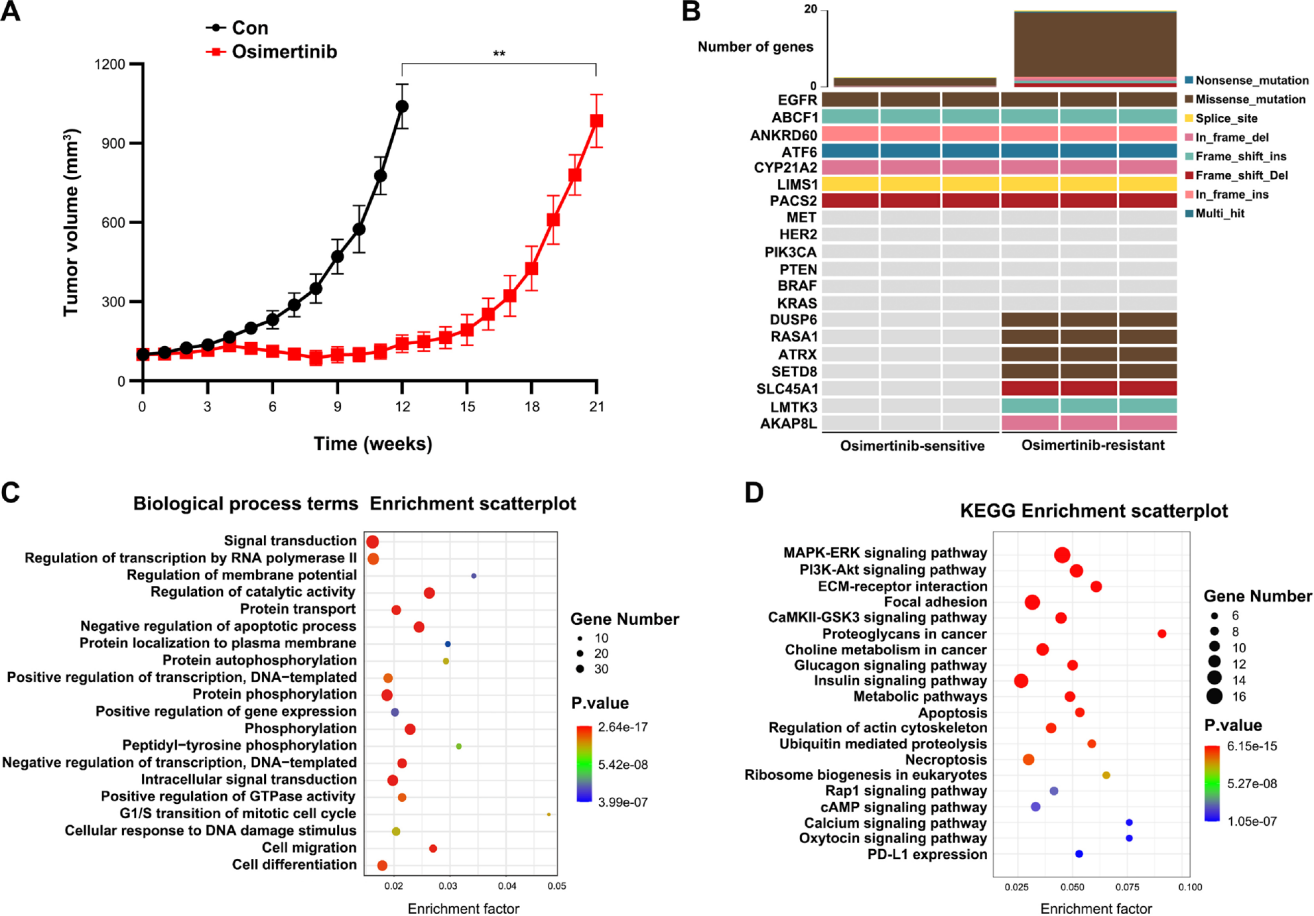
Induction of acquired resistance to osimertinib in NSCLC PDX model. **A** PDX tumor growth curves under continued treatment with Osimertinib, comparisons between two groups were evaluated by mixed-effects model, *n* = 3, **P < 0.01 (**B**) Alteration status of selected genes before and after osimertinib resistance in PDX tumors. **C**, **D** GO Biological process terms and KEGG enrichment analyses of mutant genes involved in osimertinib resistance in PDX tumors

Osimertinib is the first-line therapy for EGFR-mutated NSCLC patients. To investigate the mechanisms underlying acquired resistance to osimertinib, we constructed an osimertinib-resistant PDX model as detailed in the methods section, which was derived from a 78-year-old non-smoking female with L858R mutation in exon 21 of EGFR (Patient#42). CT scan demonstrated a mass of 4.3*3.3 cm in the right upper lobe, and the pathological diagnosis was ADC (T1cN2M0, IIIA stage). After surgery, started taking osimertinib 80 mg/day. There were no significant side effects, and the patient had a clinical complete response (Figure S3). Osimertinib has been effective for 18 months to date. Following osimertinib treatment, the PDX model exhibited a slower growth rate for tumors compared to the control group. Additionally, a reduction in tumor size was observed after four weeks. However, after about 15 weeks of continuous osimertinib treatment, tumors increased exponentially, confirming the development of acquired resistance to osimertinib (Fig. 4A). In order to explore the resistance mechanisms and alternative therapies, tumors from osimertinib-resistant and -sensitive PDXs were subjected to WES analysis. Results showed that the detected number of mutations in osimertinib-resistant PDX tumors was dramatically increased, which was about 10 times more frequent than sensitive tumors. Many wild-type genes in sensitive tumors underwent mutation during the development of osimertinib resistance, such as *dual-specificity phosphatase 6 (DUSP6)*, *Ras p21 protein activator 1 (RASA1)*, *ATRX*, *SETD8*, etc. However, the common genetic alterations involved in osimertinib resistance, such as *EGFR* C797S, *MET* amplification, and *BRAF* mutation(Cooper et al. 2022), were not detected in osimertinib-resistant PDXs (Fig. 4B). Then we performed Gene Ontology (GO) Biological process terms and Kyoto Encyclopedia of Genes and Genomes (KEGG) pathway enrichment analyses for the variant genes occurred within osimertinib-resistant PDX tumors. The results suggest that these genes were significantly clustered in the protein phosphorylation term and MAPK-ERK signaling pathway. Interestingly, both DUSP6 and RASA1 can decrease the protein phosphorylation and activity of MAPK-ERK pathway (Chen et al. 2019, 2020), and their genetic mutations might abolish their functions (Vo et al. 2019; Hayashi et al. 2018). M62I mutation of *DUSP6* was identified in all three osimertinib-resistant PDX tumors, while T846A, N850S, or I931T mutation of *RASA1* was detected in at least one osimertinib-resistant PDX tumor. Therefore, the overactivation of MAPK-ERK signaling pathway might play a pivotal role in the development of osimertinib resistance in Patient#42, and the specific inhibitors for this pathway could be considered as an alternative treatment after osimertinib failure.

### DUSP6 M62I mutation reduces osimertinib sensitivity in NSCLC

**Fig. 5:**
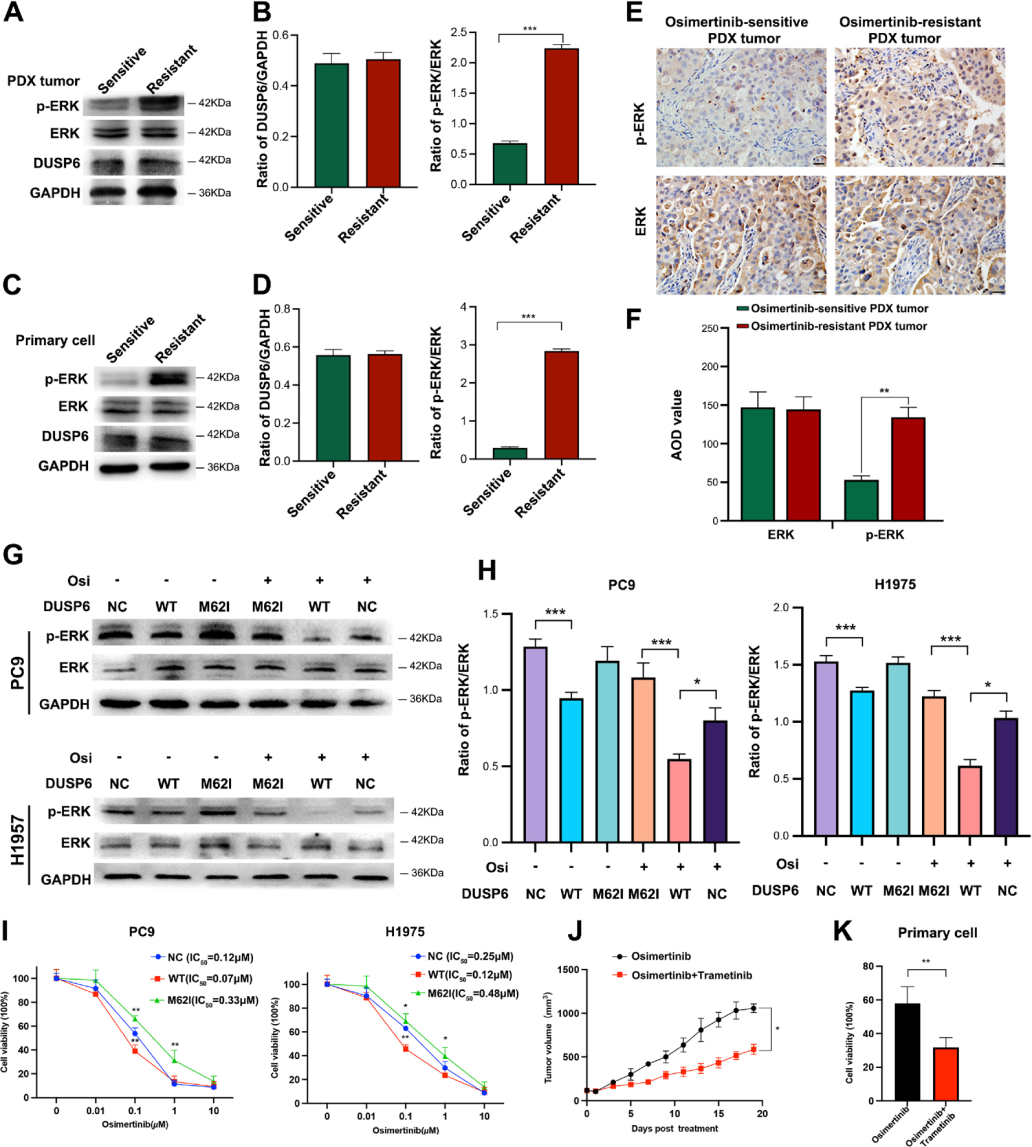
DUSP6 M62I mutation reduces drug osimertinib sensitivity in NSCLC. **A**-**D**, **E**-**F** Expression of ERK1/2 and p-ERK1/2 in osimertinib-sensitive and -resistant PDX tumors. Scale bar, 50 μm. AOD: Average Optical Density. Each group contains three tumors. From each tumor, five slices were selected from different parts, and three representative fields of view were taken from each slice. **G**-**H** The effects of osimertinib-induced ERK inhibition were examined in NSCLC cells transfected with WT DUSP6 or M62I mutant DUSP6, or a negative control (NC) plasmid. G, Representative western blot showing p-ERK and ERK expression in PC9 and H1975 cell. H, Quantification of p-ERK expression levels normalized to ERK. **I** The impacts of WT DUSP and M62I mutant DUSP on the osimertinib-induced cell viability decrease in NSCLC cells. **J** Trametinib increased osimertinib sensitivity in osimertinib-resistant PDXs mice. **K** CCK8 assays were performed to assess the primary tumor cells isolated from osimertinib-resistant PDX tumors cells treated with 500 nM of osimertinib or combination with 30 nM trametinib for 72 h. The western blot and cell viability detection were performed with three technical replicates. Comparisons between two groups were evaluated by Student’s t-test, one-way ANOVA was used to compare the means of more than two groups, *P < 0.05, **P < 0.01, ***P < 0.01

It is well known that MAPK-ERK signaling pathway plays a pivotal role in various biological events, including metabolic reprogramming, cell proliferation, survival, and differentiation (Asl et al. 2021). DUSP6, a broadly expressed dual-specificity phosphatase protein, has been assumed to bind and dephosphorylate ERK, leading to decreased ERK activity (Vo et al. 2019). In our osimertinib-resistant PDX tumors, we found a consensus missense variant, M62I, located within the ERK-binding domain of DUSP6. Previous research has suggested that M62I mutation could reduce the interaction between DUSP6 and ERK, resulting in increased ERK phosphorylation and ERK activity (Vo et al. 2019). However, it has not been evaluated so far whether the DUSP6 M62I mutation could influence the sensitivity of osimertinib. To further elucidate the activation status of the MAPK-ERK signaling pathway and the expression level of DUSP6 in osimertinib-sensitive and -resistant PDX tumors, we conducted WB analysis on PDX tumors and primary tumor cells isolated from PDX tumors. As anticipated, we observed no significant difference in DUSP6 levels between the two groups, while p-ERK levels were elevated in resistant tumors in comparison to sensitive tumors (Fig. 5A-D). IHC analysis results also showed the level of p-ERK was increased in the resistant tumors compared to the sensitive tumors (Fig. 5E-F). To further investigate the effect of the DUSP6 M62I mutation on the sensitivity of NSCLC to Osimertinib, we overexpressed wild-type (WT) DUSP6 and M62I mutant DUSP6 in PC9 and H1975 cell lines, and evaluated DUSP6 protein level using Western blot. The data showed a significant increase in DUSP6 expression after transfection, with no significant difference between the WT and M62I mutant groups (Figure S4). The results demonstrate successful overexpression of DUSP6 and suggest that the M62I mutation has no effect on DUSP6 protein expression. However, we found that the effect of osimertinib on p-ERK1/2 inactivation was dramatically enhanced after DUSP6 overexpression, while it was attenuated by M62I DUSP6 mutation (Fig. 5G-H). Moreover, we found that overexpression of DUSP6 showed a synergistic anti-viability effect with osimertinib in NSCLC cells, but DUSP6 M62I mutation significantly decreased the cellular osimertinib sensitivity. (Fig. 5I). Finally, we selected trametinib, a specific inhibitor of the MAPK-ERK signaling pathway (Han et al. 2021), to treat osimertinib-resistant PDX mice and primary tumor cells isolated from osimertinib-resistant PDX tumors, and the results showed that the tumor growth was significantly slowed down and the cell viability was statistically significant reduction after combined application of osimertinib and trametinib (Fig. 5J-K). Collectively, these data demonstrate that the DUSP6 M62I mutation-induced MAPK-ERK pathway overactivation is an important mechanism and therapeutic target of osimertinib resistance in NSCLC.

## Discussion

As one of the most lethal malignancies worldwide, NSCLC has become a paradigm of precision medicine, with the discovery of numerous subtypes defined by specific oncogenic driver mutations leading to the development of a range of molecular targeted therapies (Otano et al. 2023). During the past 20 years, more than 30 drugs have been approved by the FDA as treatments for NSCLC, including 28 targeted therapeutic drugs (Wu and Lin 2022). However, due to the continual discovery of novel driver oncogenes and the emergence of drug resistance, there is an urgent need to develop new drugs to meet clinical demands. Exploring new treatment targets and testing new treatment strategies using appropriate in vivo models should be prioritized. For traditional animal models, it is impossible to simulate all subtypes of NSCLC, but PDXs can overcome this limitation, which recapitulate faithfully many aspects of the primary tumors histology, karyotype, and genomics, as well as expected sensitivity and resistance patterns to various treatment regimens observed in patients (Woo et al. 2022).

In this study, we successfully established 13 PDXs from 62 NSCLC surgery patients, including eight ADC, four SCC, and 1 LCNEC. Consistent to previous studies, the grafting efficiency positively correlated with the histological subtype and clinical stage of the primary tumors. ADC was found to have a lower grafting efficiency compared to SCC and LCNEC, which is likely due to the inherent properties of these histologic subtypes (Pardo-Sanchez et al. 2021; Kanaki et al. 2021). Additionally, we reported here that the ability to establish PDXs was a strong marker of poor prognosis for both OS and PSF in NSCLC, which is consistent with the results from EGFR-mutant NSCLC PDXs (Stewart et al. 2015). This could be attributed to the survival period of patients and the chance of tumor formation in mice are both negatively correlated with the degree of tumor malignancy.

A large number of studies have confirmed that PDXs can preserve well the morphological and genetic characteristics of the primary tumors (Liao et al. 2023; Jung et al. 2020; Chen et al. 2021). In this study, we also analyzed the histological characteristics of the tumors by using a combination of markers that serve as diagnostic tools for the NSCLC subtype. We found that the gross morphologic and histologic features of the primary tumors were preserved in PDX tumors. But the PDX tumors were usually poorly differentiated and did not have some histology-specific structures, which is consistent with previous finding (Wu et al. 2018). This might be due to the fact that the poorly differentiated parts of patient tumors tend to be preserved and form tumors in mice, but these poorly differentiated parts can reflect drug efficacy and prognosis of the patients more accurately (Yamazaki et al. 2012; Tajima et al. 2017). In the genetic aspect, most of SNPs found in the primary tumors were retained in their matched PDXs. However, dramatically increased number of genetic alterations were detected in PDX tumors compared to their primary tumors, which might be due to the fact that when the primary tumor is growing in mice, the tumor cells need to adapt to the new host environment, leading to enhanced genomic instability and increased mutation rate. Furthermore, the discrepancy may be attributed to the higher tumor purity observed in the PDX tumors. The higher tumor purity observed in PDX tumors following the removal of mouse read contamination will facilitate the detection of genetic alterations which are at a low frequency in the primary tumor.

Next, we selected three patients (one ADC, one SCC, and one LCNEC) and their corresponding PDXs to carry out the drug experiments. The clinical responses of patients were also observed in their paired PDX models, but two targeted drugs, sotoasib and anlotinib, showed better efficacy and lower toxicity than conventional chemotherapy in the ADC and SCC PDX, respectively. In other words, the conventional chemotherapy in the clinic is not the best treatment for these two patients. However, there are limitations to the use of PDX models in cancer research. The implantation rate of PDX models for slow-growing tumors remains limited, which can make their application in personalized medicine time-consuming.

As we know, the establishment of PDX model needs a considerably long time for initial tumor engraftment, which would limit its usefulness as a director to select the first-line regimens for patients. However, successfully engrafted PDXs could be used as a co-clinical study model for patients to choose appropriate second- or third-line regimens. According to our results, these two NSCLC patients might be benefited from sotorasib or anlotinib treatments after failure of conventional chemotherapy.

In the application of PDX model, we probed into the mechanisms of acquired resistance to osimertinib. Based on the WES analysis, we identified that the variant genes in osimertinib-resistant PDX tumors were mostly enriched in protein phosphorylation term and MAPK-ERK pathway, and two mutant genes, *DUSP6* and *RASA1*, are simultaneously involved in the phosphorylation and activation of MAPK-ERK pathway. Therefore, DUSP6 is considered as a potential tumor suppressor (Kidger and Keyse 2016). Previous studies have confirmed that in NSCLC cells, knocking down DUSP6 results in enhanced ERK activation, whereas overexpressing DUSP6 leads to a decreased ERK activation and enhanced cell apoptosis (Zhang et al. 2010). In addition, DUSP6 has a synergistic effect with EGFR-TKI, and was found to be downregulated after EGFR-TKI resistance (Howell et al. 2023). We could conclude that mutations in DUSP6 could be a potential effective target for clinical treatment. Consistent with previous research, we also found that DUSP6 attenuated ERK activation and had a synergistic anti-proliferation effect with osimertinib in NSCLC cells, which was abolished by M62I mutation. Therefore, we combined trametinib, the first FDA-approved MEK inhibitor for the treatment of BRAF V600E-mutant melanoma, to treat tumors with acquired resistance to osimertinib. Both previous investigations (Jeanson et al. 2019) and our findings in this study have shown that targeting MAPK-ERK pathway with MEK inhibitors can overcome acquired resistance to osimertinib, indicating that trametinib might be an optimal choice for NSCLC patients with osimertinib resistance (Li et al. 2020; Della Corte et al. 2018; Tricker et al. 2015).

In summary, we established 13 serially transplantable PDXs for NSCLC. These PDXs recapitulated the features of the primary tumors, and the treatment responses for conventional chemotherapeutic agents in them were consistent with their corresponding patients in the clinic. Moreover, based on PDXs were excellent models, we select the optimized treatments, as well as to characterize the chemoresistance mechanisms for individual NSCLC patient, which is promising to be applied to the clinical in the future.

## Supplementary Information

Additional file 1.

## Data Availability

No datasets were generated or analysed during the current study.
