# Supplementary material for: Utility of patient-derived xenografts to evaluate drug sensitivity and select optimal treatments for individual non-small-cell lung cancer patients
Source: Mol Med. 2024 Nov 11;30:209. doi: 10.1186/s10020-024-00934-4 (PMC11556205; doi:10.1186/s10020-024-00934-4)
Supplement: Supplementary file 1 — Additional file 1. [file 10020_2024_934_MOESM1_ESM.pdf]

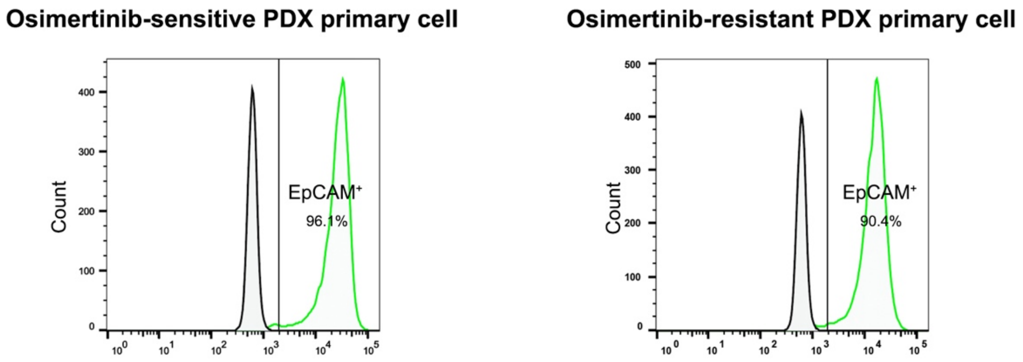

Figure S1. Establishment of patient-derived cells. Primary cultures were stained with matching isotype control (black curve) or FITC-conjugated human EpCAM antibody (green curve) and analyzed by flow cytometry. The x-axis shows fluorescence intensity and the y-axis shows the number of stained cells. A proportion of EpCAM-expressing cells were >80% in cases.

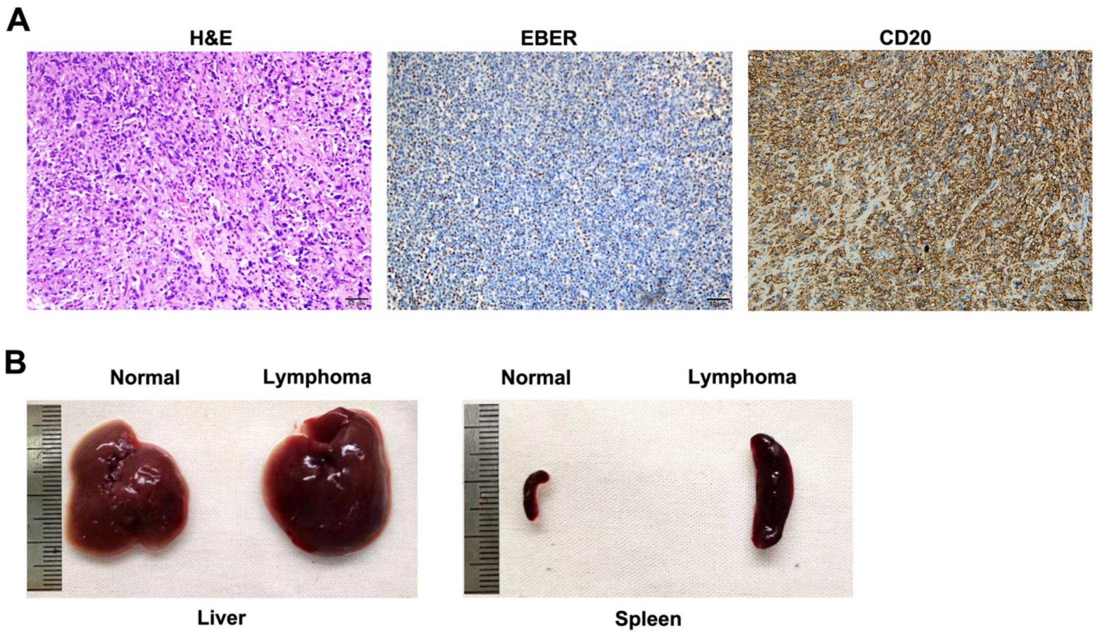

Figure S2. Representative histology of lymphoma transformed PDX tumors. (A) Representative images of H&E and EBER-ISH and IHC staining for CD20 in PDX tissues. Scale bars, 50 μm. (B) Comparison of liver and spleen between normal mice and lymphoma mice.

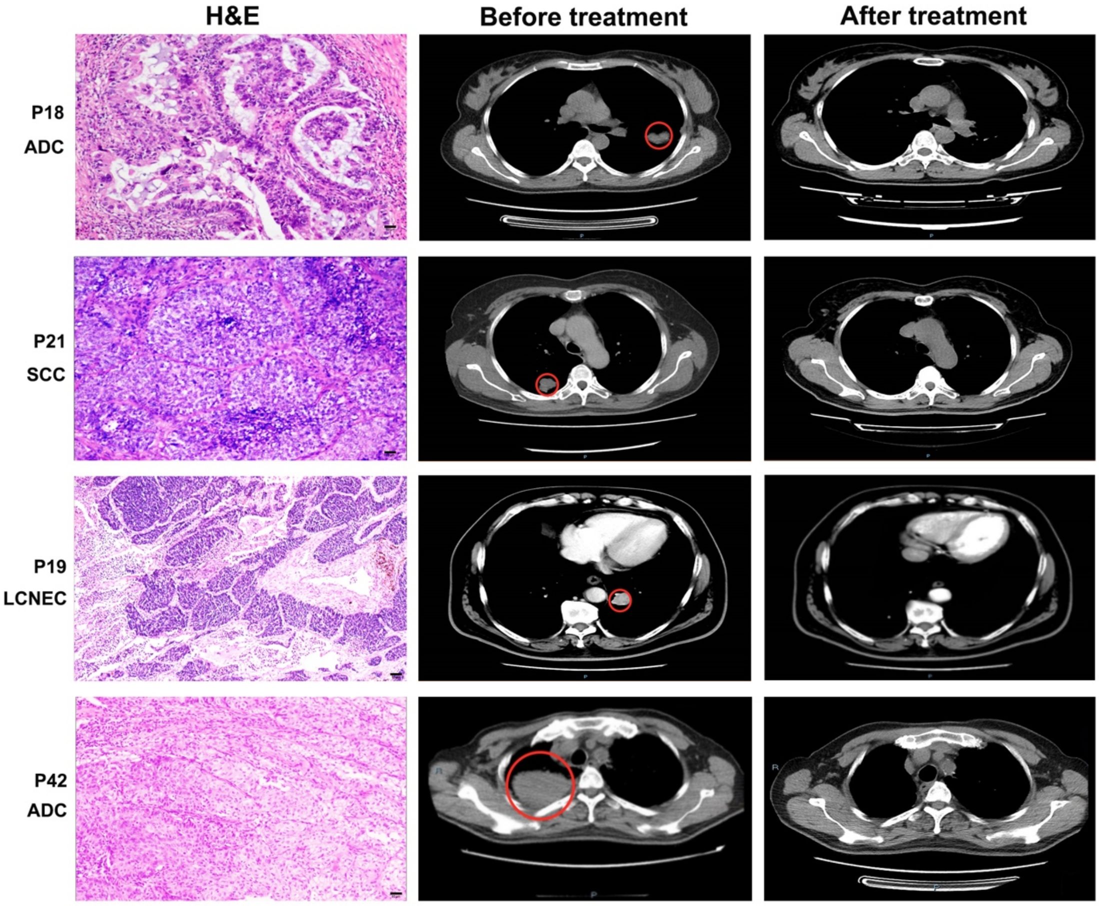

Figure S3. The histopathologic features and clinical responses of selected patients.

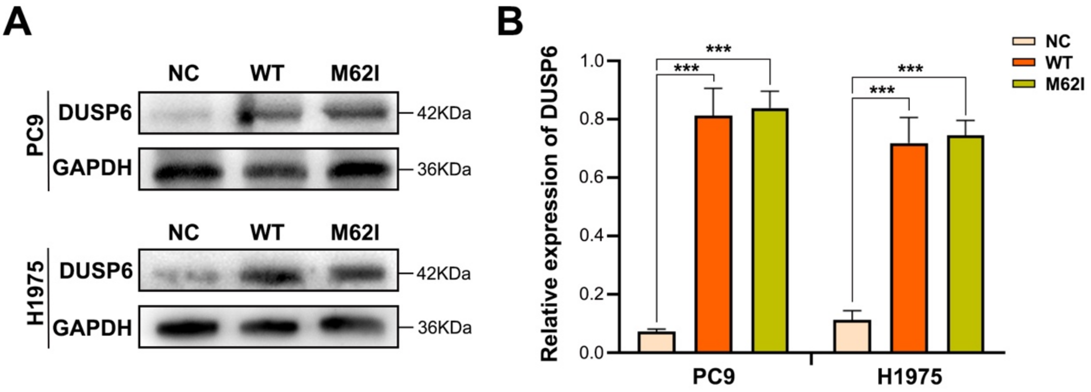

Figure S4. The protein levels of DUSP6 were assessed using immunoblot analysis. (A) Representative Western blot showing DUSP6 expression in PC9 and H1975 cell. (B) Quantification of DUSP6 expression levels normalized to GAPDH. The Western blot detection were performed with three technical replicates. Comparisons between two groups were evaluated by Student’s t-test. ***$P < 0.001$

Table S1. Clinical and pathological characteristics of 62 NSCLC patients

Patient ID	Sex	Age (years)	Smoking habits	Histology	TNM	Stage	Site	Tumor size (cm3)	Metastasis	Driver oncogene status	Engraftment success
Patient#1	Female	50	No	ADC	T2N3M1	ⅣA	LLL	13.50	Yes	ALK^{+}	Yes
Patient#2	Female	55	No	ADC	T1cN0M0	ⅠA3	LUL	1.51	No	N/A	No
Patient#3	Female	64	No	SCC	T1bN0M0	ⅠA2	RLL	1.52	No	WT	Yes
Patient#4	Male	58	No	ADC	T2bN0M0	ⅡA	RLL	3.56	No	EGFR exon 20 ins	No
Patient#5	Female	78	No	ADC	T1cN2M0	ⅢA	RLL	4.05	Yes	KRAS G13X	No
Patient#6	Female	69	No	ADC	T2aN0M0	ⅠB	LUL	4.86	No	EGFR exon 21 L858R	No
Patient#7	Female	59	No	ADC	T1cN1M0	ⅡB	LUL	5.10	Yes	EGFR exon 19 del	No
Patient#8	Female	55	No	ADC	T2aN2M0	ⅢA	RUL	5.60	Yes	EGFR exon 19 del	No
Patient#9	Male	68	Yes	ADC	T1bN2M0	ⅢA	RUL	5.78	Yes	WT	Yes
Patient#10	Female	48	No	ADC	T2aN0M0	ⅠB	LUL	6.05	No	EGFR exon 21 L858R	No
Patient#11	Male	50	Yes	ADC	T1bN0M0	ⅠA2	RUL	6.30	No	KRAS G13X	No
Patient#12	Male	64	Yes	ADC	T1bNxM0	ⅡB	RLL	6.58	Yes	EGFR exon 19 del	No
Patient#13	Female	55	No	ADC	T1cN0M0	ⅠA3	LUL	7.42	No	WT	No
Patient#14	Female	58	No	ADC	T1cN2M0	ⅢA	LUL	7.45	Yes	EGFR exon 19 del	No
Patient#15	Male	66	Yes	ADC	T1bN0M0	ⅠA2	LUL	7.50	No	WT	No
Patient#16	Female	74	No	ADC	T1cN0M0	ⅠA3	RUL	7.84	No	MET exon 14 alternative splicing aberrations	No

Patient#17	Male	70	Yes	ADC+SCC	T1cN0M0	Ⅰ A3	LUL	9.26	No	N/A	No
Patient#18	Female	69	No	ADC	T2aN2M0	ⅢA	LUL	12.80	Yes	KRAS G12C	Yes
Patient#19	male	66	Yes	LCNEC	T2aN0M0	Ⅰ B	LLL	13.50	No	WT	Yes
Patient#20	Female	69	No	ADC	T1cN2M0	ⅢA	RUL	13.85	Yes	EGFR exon 21 L858R	No
Patient#21	Male	58	Yes	SCC	T2aN0M0	Ⅰ B	RLL	14.60	No	WT	Yes
Patient#22	Male	69	Yes	ADC	T4N0M1a	ⅣA	RUL	15.01	Yes	EGFR exon 21 L858R	No
Patient#23	Male	74	No	ADC	T2aN1M0	ⅡB	RLL	15.80	Yes	WT	lymphoproliferation
Patient#24	Male	53	Yes	ADC	T1cN0M0	Ⅰ A3	RUL	16.30	No	EGFR exon 19 del	No
Patient#25	Female	54	No	ADC	T2aN0M0	Ⅰ B	RLL	16.30	No	EGFR exon 19 del	No
Patient#26	Female	51	No	ADC	T2aN0M0	Ⅰ B	RLL	17.36	No	N/A	No
Patient#27	Female	68	No	ADC	T2N2M0	ⅢA	LUL	18.00	Yes	EGFR exon 21 L858R	lymphoproliferation
Patient#28	Female	70	No	ADC	T2aN0M0	Ⅰ B	RML	18.25	No	EGFR exon 21 L858R	No
Patient#29	Male	71	No	ADC	T2bN0M0	ⅡA	LUL	19.50	No	EGFR exon 19 del	No
Patient#30	Female	56	No	ADC	T1cN0M0	Ⅰ A3	LLL	19.60	No	EGFR exon 21 L858R	Yes

Patient#31	Female	71	No	ADC	T1cN0M0	ⅠA3	LUL	19.72	No	EGFR exon 21 L858R	No
Patient#32	Female	73	No	ADC	T1cN0M0	ⅠA3	RLL	21.88	No	WT	No
Patient#33	Male	72	Yes	ADC	T2N2M1a	ⅣA	RUL	21.88	Yes	EGFR exon 21 L858R	lymphoproliferation
Patient#34	Female	54	No	ADC	T2aN0M0	ⅠB	LUL	22.05	No	N/A	No
Patient#35	Female	72	No	ADC	T2aN0M0	ⅠB	RML	22.31	No	N/A	No
Patient#36	Female	66	No	ADC	T2aN1M0	ⅡB	RLL	22.87	Yes	EGFR exon 19 del	No
Patient#37	Female	66	No	ADC	T2aN0M0	ⅠB	LUL	24.30	No	EGFR exon 19 del	No
Patient#38	Male	59	No	ADC	T1cN0M0	ⅠA3	LUL	24.70	No	WT	No
Patient#39	Male	69	No	ADC	T2aN0M0	ⅠB	LUL	25.30	No	WT	No
Patient#40	Female	65	No	ADC	T2aN0M0	ⅠB	LUL	25.40	No	EGFR exon 21 L858R	No
Patient#41	Male	59	Yes	ADC	T2aN2M0	ⅢA	LLL	26.90	Yes	EGFR exon 18 G719X	No
Patient#42	Female	78	No	ADC	T1cN2M0	ⅢA	RUL	27.20	Yes	EGFR exon 21 L858R	Yes
Patient#43	Female	56	No	ADC	T1cN0M0	ⅠA3	LUL	27.40	No	WT	Yes

Patient#44	Female	44	No	ADC	T4NXM1a	ⅣA	LUL	27.80	Yes	EGFR exon 19 del	Yes
Patient#45	Male	56	Yes	SCC	T2bN0M0	ⅡA	LLL	27.90	No	WT	No
Patient#46	Male	63	Yes	SCC	T2aN1M0	ⅡB	LLL	29.30	Yes	KRAS G13C	Yes
Patient#47	Female	68	No	SCC	T3N1M0	ⅢA	RLL	29.60	Yes	N/A	No
Patient#48	Male	60	No	ADC	T4N2M0	ⅢB	RLL	29.74	Yes	WT	No
Patient#49	Male	63	Yes	SCC	T4N0M0	ⅢA	LUL	30.34	No	WT	Yes
Patient#50	Female	59	No	ADC	T2N2M0	ⅢA	LUL	31.97	Yes	EGFR exon 21 L858R	Yes
Patient#51	Female	67	No	ADC	T2aN0M0	ⅠB	RUL	36.00	Yes	WT	No
Patient#52	Female	58	No	ADC	T2aN0M0	ⅠB	LLL	36.20	No	WT	No
Patient#53	Male	72	No	ADC	T2aN0M0	ⅠB	RLL	46.80	No	WT	No
Patient#54	Female	72	No	ADC	T1cN0M0	ⅠA3	RLL	47.80	No	HER2 exon 20 ins	No
Patient#55	Female	71	No	ADC	T2aN0M0	ⅠB	RLL	59.30	No	EGFR exon 21 L858R	No
Patient#56	Female	61	No	ADC	T2aN0M0	ⅠB	RLL	76.10	No	EGFR exon 21 L858R	No

Patient#57	Female	67	No	ADC	T1bN0M0	ⅠA2	RML	77.33	No	WT	No
Patient#58	Female	65	No	ADC	T1cN1M0	ⅡB	RLL	79.30	Yes	WT	No
Patient#59	Female	79	No	ADC	T1bN0M0	ⅠA2	LUL	101.60	No	EGFR exon 21 L858R	No
Patient#60	Male	66	Yes	SCC	T3N0M0	ⅡB	RLL	129.60	No	WT	No
Patient#61	Male	48	Yes	ADC	T1N2M0	ⅢA	RUL	217.70	Yes	EGFR exon 21 L858R	No
Patient#62	Male	70	Yes	ADC	T1cN0M0	ⅠA3	RLL	296.30	No	KRAS G13C	No

TNM, tumor node metastasis; ADC, adenocarcinoma; SCC, squamous cell carcinoma; LCNEC, Large cell neuroendocrine carcinoma; LUL, left upper lobe; RLL, right lower lobe; RML, right middle lobe; LLL, left lower lobe; RUL, right upper lobe. Driver oncogene: The American Society of Clinical Oncology (ASCO) advocates for routine mutation testing of driver genes, including EGFR, ALK, ROS1, and BRAF, in patients with non-small cell lung cancer (NSCLC). N/A, Not Available.
